# High prevalence of chronic malnutrition in indigenous children under 5 years of age in Chimborazo-Ecuador: multicausal analysis of its determinants

**DOI:** 10.1186/s12889-022-14327-x

**Published:** 2022-10-28

**Authors:** María F. Rivadeneira, Ana L. Moncayo, José D. Cóndor, Betzabé Tello, Janett Buitrón, Fabricio Astudillo, José D. Caicedo-Gallardo, Andrea Estrella-Proaño, Alfredo Naranjo-Estrella, Ana L. Torres

**Affiliations:** 1 Instituto de Salud Pública, Facultad de Medicina, Pontificia Universidad Católica del Ecuador, Av. 12 de Octubre and Roca 1076, Quito, Ecuador; 2 Centro de Investigación para la Salud en América Latina (CISeAL), Facultad de Ciencias Exactas y Naturales, Pontificia Universidad Católica del Ecuador, Quito, Ecuador; 3 Facultad de Medicina Alberto Hurtado, Universidad Peruana Cayetano Heredia. Lima Perú, San Martín de Porres, Peru; 4 Facultad de Ciencias Exactas y Naturales, Pontificia Universidad Católica del Ecuador, Matemática, Ecuador; 5 Facultad de Ciencias Humanas, Pontificia Universidad Católica del Ecuador, Geografía, Ecuador; 6 Facultad de Economía, Pontificia Universidad Católica del Ecuador, Quito, Ecuador; 7 Facultad de Enfermería, Pontificia Universidad Católica del Ecuador, Nutrición, Ecuador; 8 Posgrado de Pediatría, Facultad de Medicina, Pontificia Universidad Católica del Ecuador, Quito, Ecuador

**Keywords:** Stunting, Children, Determinants, Indigenous, Ecuador.

## Abstract

**Background:**

Despite the multiple initiatives implemented to reduce stunting in Ecuador, it continues to be a public health problem with a significant prevalence. One of the most affected groups is the rural indigenous population. This study aimed to analyze the prevalence of chronic malnutrition in indigenous children under 5 years of age and its association with health determinants, focusing on one of the territories with the highest prevalence of stunting.

**Methods:**

A cross-sectional study in 1,204 Kichwa indigenous children under the age of five, residing in rural areas of the counties with the highest presence of indigenous in the province of Chimborazo-Ecuador. A questionnaire on health determinants was applied and anthropometric measurements were taken on the child and the mother. Stunting was determined by the height-for-age z-score of less than 2 standard deviations, according to the World Health Organization´s parameters. Data were analyzed using bivariate and multivariate Poisson regression.

**Results:**

51.6% (n = 646) of the children are stunted. Height-for-age z-scores were significantly better for girls, children under 12 months, families without overcrowding, and families with higher family income. The variables that were significantly and independently associated with stunting were: overcrowding (PR 1.20, 95% CI 1–1.44), the mother required that the father give her money to buy medicine (PR 1.33, 95% CI 1.04–1.71), the father did not give her money to support herself in the last 12 months (1.58, 95% CI 1.15–2.17), mother’s height less than 150 cm (PR 1.42, 95% CI 1.19–1.69) and the child was very small at birth (PR 1.75, 95% CI 1.22–2.5).

**Conclusion:**

One out of every two rural indigenous children included in this study is stunted. The high prevalence of stunting in the indigenous and rural population is multicausal, and requires an intersectoral and multidisciplinary approach. This study identified three fundamental elements on which public policy could focus: (a) reduce overcrowding conditions, improving economic income in the rural sector (for example, through the strengthening of agriculture), (b) provide prenatal care and comprehensive postnatal care, and (c) promote strategies aimed at strengthening the empowerment of women.

**Supplementary Information:**

The online version contains supplementary material available at 10.1186/s12889-022-14327-x.

## Background

Stunting has catastrophic and permanent effects on people’s lives. It has been estimated that by 2019, 144 million children under five suffer from stunting, representing 21.33% globally [1]. Several studies have also shown that children suffering from this condition have a higher risk of death, repeated infections, and their physical, cognitive, and socio-emotional development is affected. The impact of stunting is also seen in the long term, as the development of chronic non-communicable diseases in adulthood and all these factors, not only impact the individual level, but also, the entire society with human loss and social capital [2, 3].

Stunting is particularly concentrated among poor families living in rural areas [4]. In Latin America and the Caribbean, many of these poor families belong to diverse ethnic groups, such as: indigenous, African descents or mestizos, characterized by widespread socio-economic inequality [4, 5]. In Ecuador, the prevalence of stunting in children under five has not decreased significantly in the past three decades. The survey “National Health and Nutrition Survey of Ecuador” (ENSANUT) showed a prevalence of 25.3% and 23.0%, in 2012 and 2018, respectively. Among the indigenous population, a reduction of 3.8% was observed in the prevalence of stunting between the two surveys (42.3% vs. 40.7%) [6, 7]. However, the two studies are not strictly comparable, and ENSANUT 2018 could underestimate the true prevalence of stunting. The prevalence of stunting in the indigenous population is practically double the national prevalence. As in other ethnic groups in Latin America, these populations experience greater inequalities in health, which are added to historical problems, such as dispossession of their territories and loss of their cultural and care practices. [8].

Similarly, a previous model recognizes that stunting is a multi-causal problem that is influenced by structural determinants of health, such as poverty, intermediate determinants, such as access to food, health services, among others, and immediate determinants, such as recurrence of infectious diseases and limited food intake [9]. Currently, there are gaps in knowledge in the main determinants associated with stunting in the rural indigenous population, which might allow for developing preventive policies and strategies.

The objective of this study is to analyze the determinants of stunting in the Ecuadorian indigenous population, focusing on one of the territories with the largest indigenous presence, with the purpose of guiding intersectoral responses of public and private actors involved in childcare. Maternal and child healthcare, exclusive breastfeeding and complementary feeding, accessible local food, access to health and intercultural care services, promotion of family planning and birth spacing, and implementation of stimulation and child development programs [10], are key strategies to fight the causes of stunting.

It is clear that decision makers from different sectors, such as health, social protection, education, economics, and production have responsibility for children, pregnant women, and their family’s wellbeing in order to guarantee access to poverty alleviation strategies, water, sanitation, and hygiene interventions. Therefore, the analysis of health determinants offers a theoretical framework to understand the coordinated actions between different sectors and actors. The purpose of this research is to make visible the need for an articulated, multisectoral and multidisciplinary work to respond to those determinants strongly associated to stunting.

## Methods

### Study and setting

We conducted a cross-sectional study between 2018 and 2019 in Chimborazo, Ecuador. Chimborazo is a province located in the south-central part of the country, in the Andes mountain range (average altitude 3900 m.a.s.l.). It occupies a territory of about 5,999 km², and has a population of 524,004 inhabitants [11]. 38% of the population self-identify as indigenous, placing it as one of the main indigenous territories of Ecuador [12]. Its economy is centered on the agricultural production of cereals, potatoes, vegetables, and some fruits; livestock also stands out, as well as the production of handicrafts and manufacturing such as textiles and leather. Some of the main industries of cement, ceramics, and wood are based in this province. The indigenous population of rural areas is basically dedicated to agriculture, livestock, crafts, and construction. Some residents work as day laborers planting and harvesting crops. This study was carried out in the counties of (territorial unit smaller than the province): Alausí, Guano, Guamote, Colta, and Riobamba, which hold the highest percentages of the indigenous population in the province [12].

### Study population and sample size

A sample of 1204 indigenous children, aged 0–59 months, was studied. The sample was calculated considering the population size of 14,054 indigenous children from rural areas of the counties studied, according to the 2010 National Census [11], for an expected percentage of child stunting in indigenous people of 40.7% [6], with a 95% confidence level and 3% error. Children were recruited at daycare centers and schools. Children who received treatment for infectious diseases or who were hospitalized in the two weeks prior to the survey were excluded from the study. Children with birth complications such as prematurity, congenital defects or another condition that impair growth and development were also excluded.

### Data collection procedures

We used a survey based on the Spanish version of the Questionnaire for children under five from the Multiple Indicator Cluster Survey (MICS) designed by UNICEF [13] and the National Health and Nutrition Survey of Ecuador (ENSANUT) [6, 14]. The survey includes data about demographic, socio-economic, environmental, and biological characteristics; feeding and childcare practices; and use of health services. Face-to-face interviews were conducted with the primary caregivers of the surveyed children. The information was collected by trained nutritionists.

Children and mothers were weighed on portable electronic microscales (ADE, model M320600, Hamburg, Germany). The height of mothers and children older than two years was measured with a portable stadiometer (SECA model SECA 213, Hamburg, Germany). In children under two years of age, the length of the reclining baby was obtained with a length table (model ADE MZ10027-1, Hamburg, Germany). The final measurement resulted from the mean of two measurements. Variations of 100 g in weight and 0.1 cm in height and length between the two measurements were considered acceptable. The instruments were periodically calibrated. The recommended criteria for anthropometric evaluation were followed [6]. Height-for-age Z-scores (HAZ) were calculated using 2006 WHO growth standard references [15].

### Analysis model and variable description

The dependent variable was stunting (HAZ < -2 SD), categorized into yes/no. The analysis followed a multi-causal model [16, 17], which identified basic, underlying, and immediate causes of stunting, previously used by the authors [14]. The basic causes include socioeconomic characteristics, such as lack of income and low parental education. The underlying causes refer to problems in access to food, health care, and an adequate environment; while, the immediate causes include biological characteristics, such as recurrence of infections and other variables intrinsic to the individual [14].

**Fig. 1:**
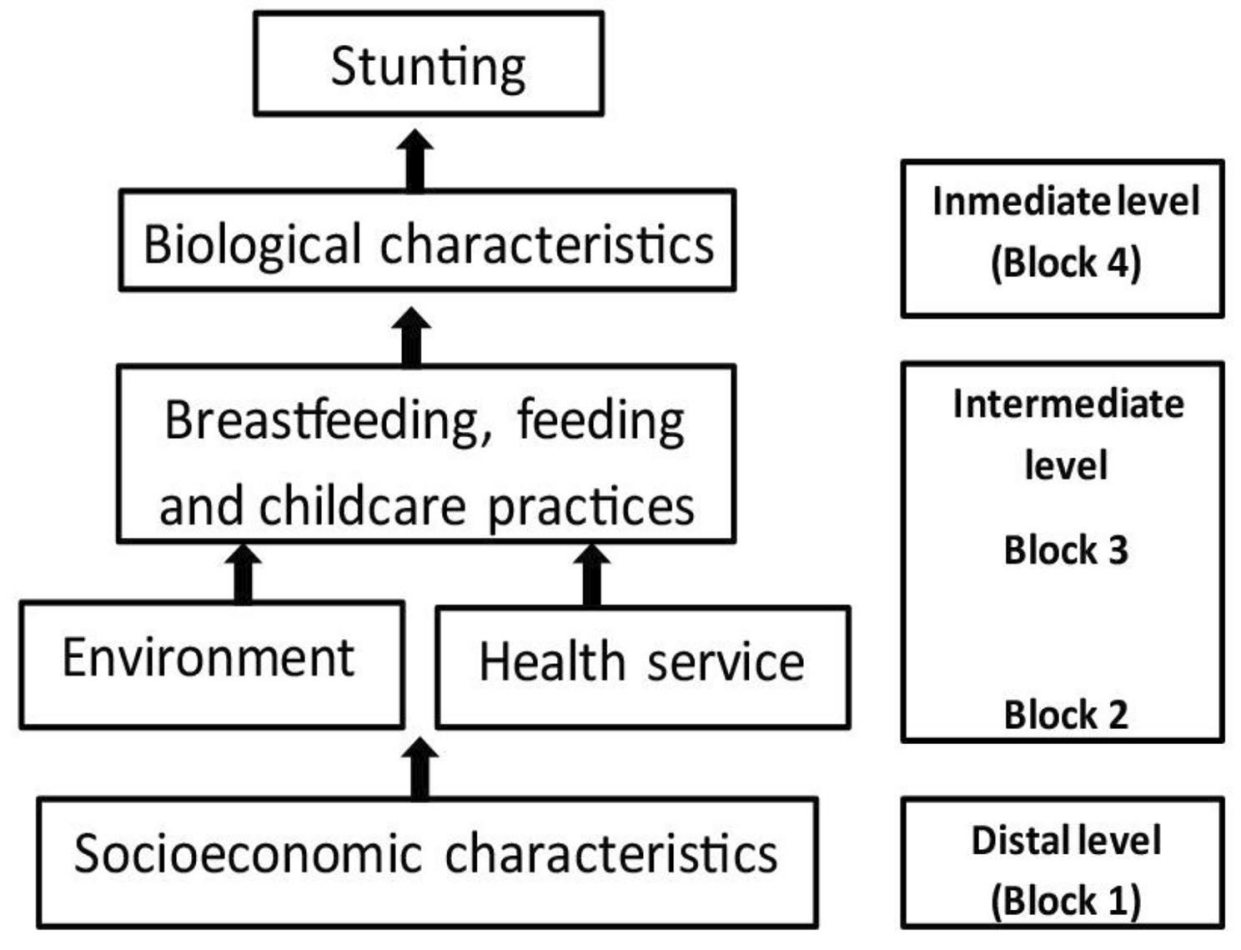
Conceptual framework for analysis of determinants associated with stunting The figure shows the Blocks: 1, 2 and 3 of analysis of the health determinants associated with stunting

From this model, the independent variables were classified into four blocks or levels of analysis (Fig. 1): Block 1, included the socioeconomic variables (family income, education of mother and father, work and housing characteristics). Block 2, the intermediate level, included the environmental characteristics (water supply, excreta and garbage disposed, and overcrowding) and variables related to health services access (proximity to the health service, place where the delivery took place, check-ups after the birth). In this case, overcrowding was defined as three or more people using the same room to sleep. Block 3 included feeding and care practices (exclusive breastfeeding in the first 6 months from birth, age at which food was introduced, food diversity or consumption of at least four food groups one day prior to the survey for children older than 6 months; practices of care included if the mother requires permission from the father to take the child to a health care facility, or requires him to give her money to buy medicine and to support himself in the last twelve months, and the daily time spent preparing food). Block 4, the immediate level, included the biological characteristics (sex, age, mother´s age, mother´s height, length of the child at birth, number of children by mother, diarrheal episodes in the last six months, and the number of episodes of parasitic infections diagnosed in the last year according to mother´s information) [14]. Because no information was available on the child’s birth, the mother was asked what the child’s length was at birth compared to other children. Based on preliminary surveys such as ENSANUT − 2012 and ENSANUT-2018, the mother was given the option to choose if her child had a birth length: ‘Very large, Average length, or Very small’, compared to other children. The option ‘Don’t know/don’t remember’ was also given for those mothers who were not sure of their answer.

### Statistical analysis

First, the characteristics of the sample and the proportion of children with stunting were described. Next, a bivariate analysis was performed on each block of explanatory variables (Fig. 1). The variables that showed a significant association with stunting, with p-values ​​less than 0.20 were kept for the multivariate analysis. The analysis was carried out according to the methodology proposed by Victora et al., 1997 [16], and Poisson regression models (Prevalence Ratio and 95% CI) were used in multivariate analysis. In each block, the statistically significant variables were maintained (p < 0.10) for the subsequent stages. The procedure began with Block 1, of socioeconomic variables. Then, for the second stage, Block 2, of environmental and health services variables were included in the model. In the third stage, the variables from Block 3 of breastfeeding, feeding, and care were added. Finally, the variables from Block 4, of biological characteristics, were added. For the final model, all the variables that were significant in the previous stages were taken and only those that were statistically significant were kept (p < 0.05) [16].

## Results

### Sample characteristics

A total of 1251 children were invited to participate in the study, of which 1204 children (96.2%) had complete data and were included in the analysis. Table 1 characterizes the study population. The socioeconomic characteristics show that 35.1% (n = 397) belong to the lowest income quintile, a higher percentage of children whose parents have basic education (57.93% mother and 48.42% father); and 57.77% children with unemployed mothers. It should be considered that unemployed women in the rural sector dedicate their full time to agricultural work and housekeeping. According to the household characteristics, 56.33% have potable water, 38.15% (n = 449) have a toilet connected to the sewage system, and 52.2% (n = 596) live in overcrowded conditions.

Regarding their biological characteristics, 50.08% (n = 603) were male, 30.65% (n = 369) were 49–86 months old, 41.59% (n = 447) were born from mothers aged 13–25 years, and 22.17% (n = 266) were very small at birth, as reported by their mothers. Other characteristics of the sample like access to health services, breastfeeding and care practices are shown in Table 1. All the studied variables are included in Supplementary Material 1.

### Stunting prevalence

**Fig. 2:**
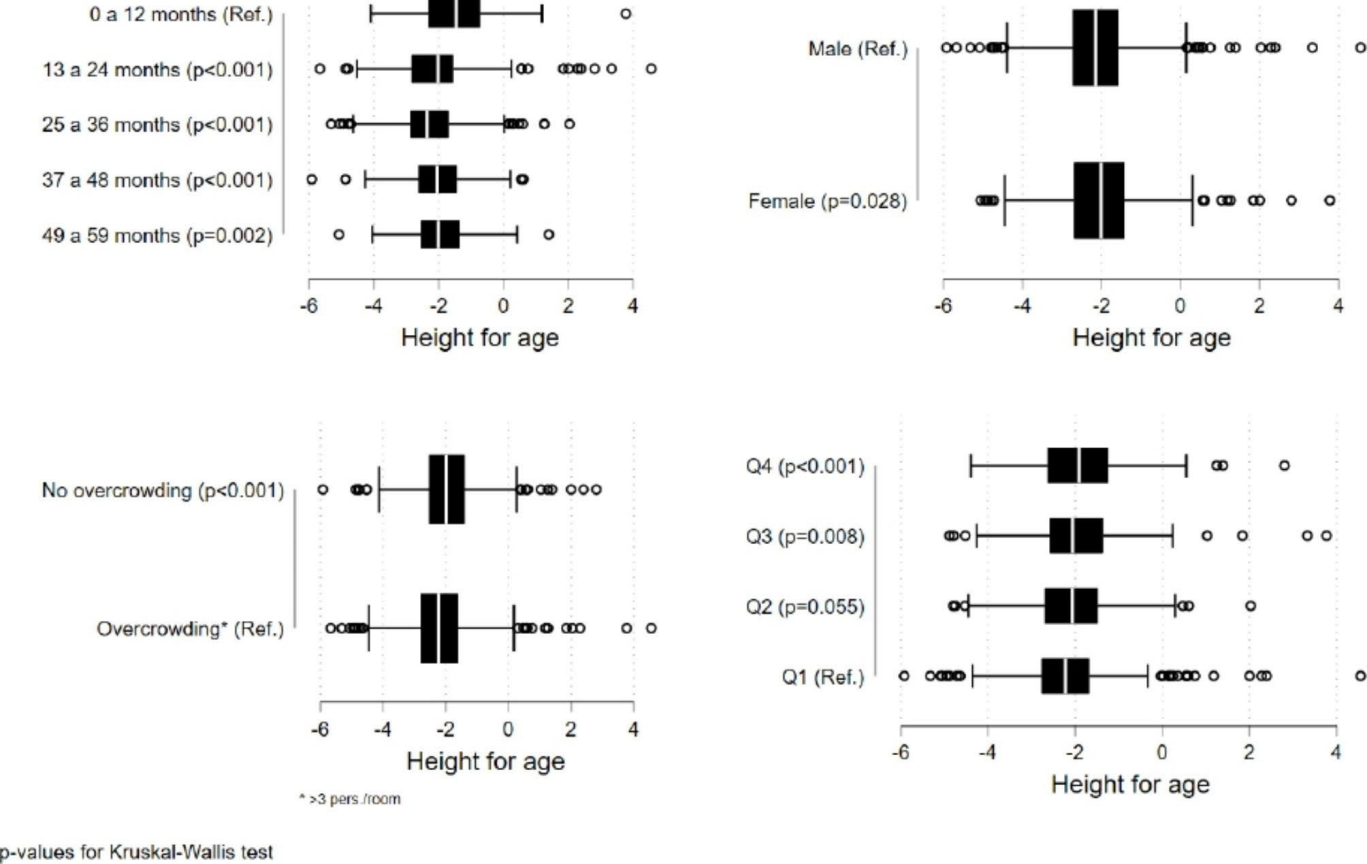
**Box-plot of height-for-age Z-scores (HAZ) according to health determinants** The results were stratified by sex, age group, overcrowding, and economic quintile. Indigenous children under 5 years of age, Chimborazo-Ecuador

Sample stunting prevalence was 51.6% (n = 646). Figure 2 presents Z-scores de HAZ by sex, age group, overcrowded conditions, and household income. Significant differences were found within medians with better scores for women, children under 12 months, families without overcrowding and families with higher family income (quintile 4).

### Health determinants associated to stunting

Table 1 shows the results of the bivariate analysis between the characteristics of the children studied and the prevalence of stunting that were statistically significant. The other variables are included in Supplementary Material 1. Regarding the socioeconomic determinants, the children in the lowest quintile (quintile 1) had a significantly higher prevalence of stunting (PR 1.27, 95% CI 1.1–1.48), than the children with the highest economic income (quintile 4). The children with mothers with elementary and primary education had a significantly 1.97 and 1.66 times higher prevalence of stunting, respectively, than the children with mothers with higher education (95% CI 1.29–3.01; 1.17–2.34, respectively). The children with parents without any initial instruction and with basic instruction presented 1.56 and 1.43 times higher prevalence of stunting respectively, compared to the children with parents with higher education (95% CI 1.14–2.15; 95% CI 1.11–1.85, respectively). Children with mothers who had worked had a significantly 1.15 times higher prevalence of stunting (95% CI 1-03-1.27) than children with mothers who were not working at the time of the survey. Regarding environmental determinants, children who received piped water located outside the house and those who did not receive piped water had a significantly 1.24 and 1.37 times higher prevalence of stunting compared to those who received piped water inside the house (95% CI 1.11–1.39; 95% CI 1.04–1.78). When the toilet was connected to a cesspool, children had a 1.21 times higher prevalence of stunting compared to those who had a toilet connected to the public sewer network (95% CI 1.05–1.4). The children whose families burn or bury garbage, the prevalence of stunting was higher than in those children whose families have public garbage collection service (PR 1.13, 95% CI 1.01–1.27). Children who live in crowded conditions have a significantly 1.22 times higher prevalence of stunting than those who do not live in crowded conditions (95% CI 1.10–1.36).

**Table 1:** Characteristics of the children included in the study and association with stunting. Bivariate regression (n = 1204)

	Sample	Stunting				
	**n (%)**	**n (%)**	**PR (95% CI)** ^**†**^		**PR (95% CI)** ^**††**^	
**Socioeconomic characteristics**						
Family income						
Q4 – highest	287 (25.38)	133 (46.34)	1.0		1.0^a^	
Q3	170 (15.03)	87 (51.18)	1.1 (0.91–1.33)		1.08 (0.8–1.46)	
Q2	277 (24.49)	151 (54.51)	1.17 (0.99–1.38)		1.07 (0.82–1.4)	
Q1 – lowest	397 (35.1)	235 (59.19)	1.27 (1.1–1.48)**		1.09 (0.84–1.42)	
Mother’s schooling level						
University/College	64 (5.8)	22 (34.38)	1.0		1.0^a^	
High-School	372 (33.73)	177 (47.58)	1.38 (0.97–1.97)		1.28 (0.77–2.12)	
Primary	639 (57.93)	365 (57.12)	1.66 (1.17–2.34)**		1.38 (0.83–2.29)	
Elementary	28 (2.54)	19 (67.86)	1.97 (1.29–3.01)**		1.40 (0.65–2.98)	
Father’s schooling level						
University/College	91 (8.22)	37 (40.66)	1.0		1.0^a^	
High-School	422 (38.12)	204 (48.34)	1.18 (0.91–1.55)		1.06 (0.7–1.59)	
Primary	536 (48.42)	313 (58.4)	1.43 (1.11–1.85)**		1.16 (0.76–1.75)	
Elementary/None	58 (5.24)	37 (63.79)	1.56 (1.14–2.15)**		1.46 (0.84–2.53)	
Mother works						
No	632 (54.77)	319 (50.47)	1.0		1.0^a^	
Yes	522 (45.23)	303 (58.05)	1.15 (1.03–1.27)*		1.18 (0.98–1.43)	
Main roof material						
Concrete	328 (27.84)	150 (45.73)	1.0		1.0^a^	
Asbestos (Eternit)	415 (35.23)	242 (58.31)	1.27 (1.1–1.47)**		1.16 (0.92–1.48)	
Zinc	327 (27.76)	174 (53.21)	1.16 (0.99–1.35)		0.99 (0.76–1.32)	
Tile/straw/other	108 (9.17)	66 (61.11)	1.33 (1.1–1.61)**		1.04 (0.72–1.51)	
Main floor material						
Parquet/ceramic/floor tile/marble	132 (11.21)	62 (9.81)	1.0		1.0^a^	
Cement/Wood	711 (60.36)	365 (57.75)	1.09 (0.89–1.32)		1.10 (0.81–1.52)	
Dirt	335 (28.44)	205 (32.44)	1.30 (1.06–1.59)**		1.18 (0.82–1.69)	
**Environmental**						
The water you receive is:						
Piped inside the house	499 (42.72)	239 (47.9)	1.0		1.0^b^	
Piped outside the house, but inside the lot	596 (51.03)	355 (59.56)	1.24 (1.11–1.39)**		1.18 (0.98–1.42)	
Piped outside the lot	41 (3.51)	13 (31.71)	0.66 (0.41–1.04)		0.47 (0.22–1.03)	
No piped water	32 (2.74)	21 (65.63)	1.37 (1.04–1.78)*		1.38 (0.75–2.56)	
The sanitary areas of the dwelling are						
Toilet connected to public sewerage system	449 (38.15)	225 (50.11)	1.0		1.0^b^	
Toilet connected to septic tank	366 (31.1)	192 (52.46)	1.04 (0.91–1.19)		0.99 (0.8–1.24)	
Toilet connected to cesspool	221 (18.78)	135 (61.09)	1.21 (1.05–1.4)**		1.06 (0.82–1.36)	
Latrine	63 (5.35)	33 (52.38)	1.04 (0.81–1.34)		0.94 (0.6–1.47)	
No sanitary area	78 (6.63)	47 (60.26)	1.2 (0.98–1.47)		0.95 (0.64–1.41)	
How is garbage disposed						
Public collection service	813 (69.37)	418 (51.41)	1.0		1.0^b^	
Dumped in the street, ravine, river	10 (0.85)	6 (60)	1.16 (0.7–1.94)		1.12 (0.5–2.54)	
Burned, buried	349 (29.78)	204 (58.45)	1.13 (1.01–1.27)**		1.06 (0.86–1.3)	
Overcrowding						
No	568 (48.8)	274 (48.24)	1		1.0	
Yes	596 (51.2)	353 (59.23)	1.22 (1.10–1.36)**		1.18 (1.08–1.41)*	
**Healthcare**						
Where did you give birth						
Health facility (public or private)	800 (70.24)	396 (49.5)	1.0		1.0^b^	
At home with midwife, family member or alone	334 (29.32)	200 (59.88)	1.21 (1.08–1.35)**		1.12 (0.42–2.97)	
Other	5 (0.44)	4 (80)	1.61 (1.03–2.51)*		1.56 (0.45–5.46)	
First check-up after birth						
Less than 1 week	290 (26.39)	140 (48.28)	1.0		1.0^b^	
1 week	201 (18.29)	106 (52.74)	1.09 (0.91–1.3)		1.03 (0.72–1.49)	
2 or more weeks	584 (53.14)	313 (53.6)	1.11 (0.96–1.27)		1.11 (0.83–1.48)	
No control	24 (2.18)	18 (75)	1.55 (1.19–2.01)**		1.50 (0.34–6.67)	
How long does it take to get to the health center?						
Less than 15 min	362 (30.78)	177 (48.9)	1.0		1.0^b^	
15–30 min	493 (41.92)	257 (52.13)	1.06 (0.93–1.22)		1.01 (0.69–1.49)	
31–60 min	223 (18.96)	135 (60.54)	1.23 (1.06–1.43)**		1.07 (0.67 − 1.69)	
More than one hour	98 (8.33)	62 (63.27)	1.29 (1.07–1.55)**		0.98 (0.44–2.17)	
**Breastfeeding and Care Practices**						
To take your kid to a health facility, you ask the father for permission.						
No	784 (69.26)	400 (51.02)	1.0		1.0^c^	
Yes	348 (30.74)	203 (58.33)	1.14 (1.02–1.27)*		1.09 (0.88–1.35)	
To buy medicines for your kid, you need money from the father						
No	304 (26.97)	148 (48.68)	1.0		1.0^c^	
Yes	823 (73.03)	451 (54.8)	1.12 (0.98–1.28)		1.33 (1–1.76)*	
The father gave you money to support the kid on the last 12 months						
Yes	990 (88.63)	516 (52.12)	1.0		1.0^c^	
No	127 (11.37)	79 (52.2)	1.19 (1.03–1.38)*		1.68 (1.19–2.38)**	
Daily time spent preparing food						
More than 120 min	623 (53.29)	315 (50.56)	1.0		1.0^c^	
From 61 to 119 min	123 (10.52)	63 (51.22)	1.01 (0.83–1.22)		0.97 (0.71–1.33)	
Up to 60 min	423 (36.18)	250 (59.1)	1.16 (1.04–1.30)**		1.02 (0.82–1.27)	
Exclusive breastfeeding						
Yes	1044 (86.71)	552 (52.87)	1.0			
No	160 (13.29)	94 (58.75)	1.11 (0.96–1.28)			
Introduction to food						
At 6 months	599 (51.86)	321 (53.59)	1.0			
More than 6 months	429 (37.14)	226 (52.68)	0.98 (0.87–1.10)			
Before 6 months	127 (11)	72 (56.69)	1.05 (0.89–1.25)			
Food diversity (6 to 23 months)						
≥ 4 food groups	206 (83.4)	99 (48.06)	1.0			
≤ 4 food groups	41 (16.6)	18 (43.09)	0.91 (0.62–1.32)			
**Biological characteristics**						
Sex						
Male	603 (50.08)	344 (57.05)	1.0		1.0^d^	
Female	601 (49.92)	302 (50.25)	0.88 (0.79–0.97)*		0.90 (0.74–1.08)	
Age (months)						
0–12	61 (5.07)	21 (34.43)	1.0		1.0^d^	
13–24	229 (19.02)	115 (50.22)	1.45 (1-2.11)*		1.52 (0.78–2.97)	
25–36	334 (27.74)	215 (64.37)	1.86 (1.31–2.66)**		1.99 (1.04–3.81)	
37–48	211 (17.52)	110 (52.13)	1.51 (1.04–2.19)*		1.66 (0.85–3.25)	
49–59	369 (30.65)	185 (50.14)	1.45 (1.01–2.08)*		1.58 (0.82–3.05)	
Mother’s age						
13–25	477 (41.59)	234 (49.06)	1.0		1.0^d^	
26–35	444 (38.71)	250 (56.31)	1.14 (1.01–1.29)*		1.07 (0.85–1.36)	
>36	226 (19.7)	129 (57.08)	1.16 (1.00-1.34)*		0.90 (0.64–1.29)	
Mother’s height						
>= 150 cm	635 (52.74)	284 (44.74)	1.0		1.0^d^	
<150 cm	569 (47.26)	362 (63.62)	1.42 (1.27–1.58)**		1.39 (1.15–1.68)**	
Birth length						
Very large	139 (11.58)	52 (37.41)	1.0		1.0^d^	
Average length	756 (63)	393 (51.98)	1.38 (1.11–1.74)**		1.41 (1–1.99)	
Very small	266 (22.17)	175 (65.79)	1.75 (1.39–2.21)***		1.68 (1.15–2.45)**	
Don’t know/don’t remember	39 (3.25)	24 (61.54)	1.64 (1.18–2.28)**		1.28 (0.45–3.63)	
Number of children by mother						
1–2	683 (58.58)	326 (47.73)	1.0		1.0^d^	
3–4	315 (27.02)	180 (57.14)	1.19 (1.05–1.35)**		1.06 (0.82–1.39)	
≥5	168 (14.41)	116 (69.05)	1.44 (1.27–1.64)***		1.32 (0.91–1.92)	
Diarrhea in the last 6 months						
None	529 (43.97)	269 (50.85)	1.0		1.0^d^	
1 to 2 times	371 (30.84)	183 (49.33)	0.97 (0.84–1.11)		0.90 (0.71–1.15)	
More than 2 times	277 (23.03)	178 (64.26)	1.26 (1.11–1.42)***		1.10 (0.85–1.42)	
Don’t know	26 (2.16)	16 (61.54)	1.21 (0.88–1.65)		1.19 (0.52–2.75)	
Times child has had parasites in the last year						
None	621 (68.62)	300 (48.31)	1.0		1.0^d^	
1 to 2 times	262 (28.95)	159 (60.69)	1.25 (1.10–1.42)**		0.94 (0.72–1.22)	
More than twice	22 (2.43)	17 (77.27)	1.59 (1.25–2.03)**		0.87 (0.66–1.16)	

^†^Non-adjusted PR (Prevalence Ratio) and 95% confidence interval (95% CI)

^††^ PR adjusted and 95% confidence interval

^a^ PR adjusted for the variables family income, mother’s schooling level, father’s schooling leves and main floor material

^b^ PR adjusted for the variables listed in ^a^ plus environmental and healthcare variables

^c^ PR adjusted for the variables listed in ^b^ plus breastfeeding and care practices variables

^d^ PR adjusted for the variables listed in ^c^ plus biological characteristics variables

^*^ significant differences (p < 0.05); ^**^significant differences (p < 0.01); ^***^significant differences (p < 0.001)

When analyzing the characteristics related to health services, it was found that children who were born at home or in other places that were not health facilities, had, respectively, 1.21 and 1.61 times higher prevalence of stunting than those who were born in health facilities, statistically significant association (95% CI 1.08–1.35; 95% CI 1.03–2.51, respectively). Children who did not receive any well-baby checkups with a health center after birth had a significantly 1.55 times higher prevalence of stunting than those who received their first control within the first week of being born (95% CI 1.19–2.01). Children who are farther from the nearest health service, 31–60 min and more than 1 h, presented 1.23 and 1.29 times higher prevalence of stunting respectively, than those who reside less than 15 min from the health service (95% CI 1.06–1.43; 95% CI 1.07–1.55).

Regarding breastfeeding and care practices, children whose mother needs to request permission from the father to take the children to a health facility had a 1.14 times higher prevalence of stunting, compared to those who do not require the father’s permission (95% CI 1.02–1.27). Likewise, the children with mothers who have not received money from the father of the child to support themselves in the last 12 months, presented 1.19 times significantly higher prevalence of stunting than those who received money from their parents to support themselves (95% CI 1.03–1.38 ). When the time to prepare food at home was reduced to less than 60 min a day, children had a 1.16 times higher prevalence of stunting than those where the time to prepare food was greater than 120 min (95% CI 1.04–1.30). No significant differences were found in terms of breastfeeding and complementary feeding indicators.

In relation to the biological determinants, on one hand, it was observed that the female sex had a significantly lower prevalence of stunting than the male sex (PR 0.88, CI 95% 0.79–0.97). On the other hand, it was observed that the prevalence of stunting increased as the age of the children advanced. Children with mothers aged 26–35 years and older than 35 years, presented 1.14 and 1.16 times greater probability of stunting than children born from mothers under 25 years of age (95% CI 1.01–1.26; 95% CI 1.00-1.34, respectively). Children born very small at birth, according to the mother’s reference, were 1.75 times more likely to be stunted than children who were very large at birth, statistically significant (95% CI 1.39–2.21). Likewise, when the mother was less than 150 cm tall, the probability that the child was stunted was 1.42 times greater than when the mother was 150 cm tall or greater (95% CI 1.27–1.58). If the mother had three or more live children, the prevalence of stunting was significantly higher compared to those children whose mothers had fewer than three live children (PR 1.19, 95% CI 1.05–1.35; PR 1.44, 95% CI 1.27–1.64 for 3–4 and 5 or more children born alive, respectively). When the child had more than two episodes of diarrhea in the last six months or more than two episodes of parasitosis in the last year, the prevalence of stunting was significantly higher, compared to those who did not have any episode (PR 1.26, 95% CI 1.11–1.42; PR 1.59, 95% CI 1.25–2.03, respectively).

### Multivariate model to stunting and health determinants

When applying the multivariate analysis by blocks, it was found that the following variables were significantly and independently associated with stunting (Table 2): living in a crowded house (PR 1.20, 95% CI 1–1.44), the mother requires that the father gives her money to buy medicines (PR 1.33, 95% CI 1.04–1.71), the father did not give her money to support herself in the last 12 months (1.58, 95% CI 1.15–2.17), mother’s height (less than 150 cm) (PR 1.42, 95% CI 1.19–1.69) and the child was very small at birth (PR 1.75, 95% CI 1.22–2.5).

**Table 2:** Hierarchical multivariate model for stunting in indigenous children under 5 years of age, Chimborazo-Ecuador (n = 1204)

	Stunting PR (95% CI) ^†^	p value	
Overcrowding			
No	1.0		
Yes	1.20 (1–1.44)*	0.047	
The mother needs money from the father when the infant is sick			
No	1.0		
Yes	1.33 (1.04–1.71)*	0.025	
The father gave money to support the kid on the last 12 months			
Yes	1.0		
No	1.58 (1.15–2.17)**	0.004	
Mother’s height			
>= 150 cm	1.0		
<150 cm	1.42 (1.19–1.69)***	0.000	
Birth length			
Very large	1.0		
Average length	1.41 (1.01–1.97)*	0.043	
Very small	1.75 (1.22–2.5)**	0.002	
Don’t know/don’t remember	1.66 (0.84–3.28)	0.142	

^**†**^PR (95% CI) = Prevalence Ratio and 95% Confidence Interval

^*^ significant differences (p < 0.05)

^**^significant differences (p < 0.01)

^***^significant differences (p < 0.001)

## Discussion

This article analyzes the health determinants associated with stunting in indigenous children under 5 years old who lived on rural areas from Chimborazo, one of the areas with the highest prevalence of stunting in Ecuador and with the largest indigenous presence. This research found that one of two indigenous children are stunted. This result exceeds national data from preliminary studies [6, 7], as well as data reported in South Asia and Subsaharian Africa considered the regions with the highest prevalence around the world, where one in three children are stunted [17]. Other studies conducted on indigenous populations have reported higher percentages of stunting compared to other ethnic groups [4, 17–20].

In Ecuador, like in other regions, stunting has an indigenous face. Despite the reduction of poverty and the improvement in the living conditions of the population in general, indigenous populations have been historically excluded, and currently live in conditions of economic and social inequality. For Latin America, ethnicity is synonymous of economic and social status, and has an important burden in the intergenerational transmission of stunting [4]. The results of this study make visible a health problem in the indigenous child population that is synonymous with poverty. On the other hand, it has been observed that, among all the determinants related to stunting, one of those that consistently predicts an increase in HAZ is the increase in family income or asset index [1]. Analytical models conducted in other countries suggest that the increase in income was responsible for 25 to 40% of the increase in HAZ [21, 22]. In addition, some studies in Brazil and Ecuador have demonstrated a strong effect of cash transfer programs on the reduction of childhood mortality from poverty-related diseases, including malnutrition [23, 24].

In this study, we found a significant relationship between stunting and environmental determinants, such as overcrowding, although this variable could also be considered as a proxy variable of socioeconomic level and an indicator of poverty. Other studies have also confirmed the relationship between worse sanitary conditions and higher prevalence of stunting; thus, limited access to safe water, lack of sewerage for excreta disposal and inadequate garbage disposal, among others, are conditioning factors of stunting [25, 26]. Both, the absence of these services and overcrowding, determine a greater risk to the presence of diseases, mainly diarrhea and infectious diseases that lead to weight and height detriments in children. Interventions aimed at improving water or excreta disposal systems have been found to predict improvements in HAZ by 7–14% [22, 27].

On the other hand, we observed that if the mother needed to ask the father for money to buy medicine or if the father did not give her money to support the household in the last twelve months, the probability of stunting in the child increased. These variables would be related to care and parenting practices, as well as to economic conditions and the female empowerment. Other authors mention that a good parental relationship is protective of child stunting, as it translates into better child care and attention practices [28, 29]. In recent years, the importance of the father’s role in child nutrition has been recognized [30]. For example, one study found that fathers’ financial contributions to children’s nutrition and health care improved their children’s nutrition [31]. Similarly, the nutritional status of Mexican American children was favorably related to father participation in feeding practices [32]. However, we consider that in our study, these variables also reflect the situation of single mothers, in unstable working conditions, with limited social support, who depend on their partners or the fathers of their children to access health care, to buy food and to pay services. Therefore, these mothers and their children would be in conditions of greater social and financial vulnerability. Previous studies have shown that the empowerment of mothers in decision-making regarding their children and a better economic status is related to a better nutritional status of the child [33, 34].

In our study, short maternal height, less than 150 cm, was significantly associated with stunting. Short maternal height has been associated with a negative effect on children’s growth [1]. This association also evidences the intergenerational burden of stunting as an effect of poverty, beyond a simple genetic or hereditary factor. Previous studies suggested that the impact of stunting extends to the next generation of children, with effects not only on height, but also on cognitive development, implying an additional impact of stunting on the economic and social development of countries [34]. These findings underline the importance of ensuring an adequate nutritional and health status of women, even prior to pregnancy [1, 35].

Children whose length was very small at birth, as perceived by their mothers, had a higher prevalence of stunting than those with a very large length at birth. Similar results were reported by other studies [25, 26]. This data would probably relate to intrauterine growth restrictions due to the lack of nutrients needed for pregnancy, which would contribute to alterations in growth during childhood. Several studies have found that improving birth weight or height is significantly associated with better infant growth [36, 37], which shows the importance of prenatal and nutritional care of the mother in the prevention of stunting.

One of the challenges for countries with a high prevalence of stunting is the definition of articulated policies and strategies to reduce child malnutrition. We have previously mentioned that stunting is a multi-causal and complex problem, influenced by structural determinants such as poverty, intermediate determinants such as food access, care and health services access, among others, and immediate determinants such as maternal height, height at birth, presence of diseases and infections, etc. The identification of these determinants is vital for those countries where the burden of stunting remains unacceptably high [1, 14]. Through the analysis presented in this research, we have proposed a model based on health determinants to guide decision-making aimed to reduce stunting in rural, indigenous populations, as in the case of Chimborazo in Ecuador. We have observed that an intersectoral and multidisciplinary action approach is necessary to respond to the determinants that condition malnutrition in this population. A key factor is governance including concrete incentives for action and joint work of sectors linked to health, economy, agricultural production, social welfare and food security, through different local and national actors. Policies and strategies should allow continuous accompaniment and care for child and mother, before pregnancy, going through gestation, and ensuring access to health benefits during the life cycle. Special attention should be given to policies favoring the parental role in childcare and the empowerment of women [33, 38]. At the same time, other policies and strategies to improve living conditions for indigenous populations are related to decreasing socioeconomic gaps that determine greater poverty and overcrowding. Interventions in this sense should be aimed at improving the economic income of the rural indigenous population through more equitable production systems, for example through state loans or bonds that strengthen local agriculture, and fairer marketing systems.

This study has several limitations. The sample was taken mainly from children attending child care and early education centers in rural areas of Chimborazo, so that the sample predominantly represents institutionalized children. This type of study, cross-sectional, does not allow establishing cause-effect relationships, but analyzes associations between determinants and stunting. The results of this study could be inferred to other indigenous populations of the Ecuadorian highlands but not to indigenous populations of the Amazon in Ecuador due to cultural differences. Some variables, such as the weight and length of the children at birth were not available for this study, since the mothers did not have this information. Another difficulty is related to the evaluation of feeding practices; here we used complementary feeding indicators related to food intake in the last 24 h as a proxy to analyze the intake and characteristics of breastfeeding and complementary feeding.

One of the strengths of the study is the analysis of the determinants associated with stunting based on a multicausal model, as well as the large number of variables included in the analysis that allow a broad approach to this problem. Another strength is the identification of children in hard-to-reach rural areas, who are usually not taken into account in the definition of public policies. At the same time, this study is based on the 5 municipalities that concentrate most of the indigenous population of Chimborazo. As already mentioned, and as other authors have questioned, stunting is a secondary problem to a multiplicity of factors and pathways ranging from the biological to the social, which are almost impossible to interrupt with isolated interventions, thus requiring profound social changes that can be extended and sustained for decades [38, 39].

## Conclusion

In this study, one out of every two indigenous children studied are stunted. The prevalence of stunting found is an alarm for all authorities at different levels of government and for organizations and institutions involved in child nutrition and rights. The findings suggest the urgent need to implement efficient intersectoral and multidisciplinary actions that prioritize rural indigenous communities. The determinants that were independently associated with stunting were overcrowding, the mother requiring the father to give her money when she needs to buy medicine, the mother not having received economic support from the father in the last twelve months, the mother’s short height, and the child’s height at birth. These results show the intergenerational transmission of stunting and the need to access prenatal and postnatal controls that guarantee compliance with health benefits, as well as to improve the living conditions of indigenous populations and strengthen the empowerment of women and the paternal role in childcare.

This study is a call for collective action regarding population interventions. It reiterates that the Ecuadorian central highlands, rural areas and the indigenous population are three critical conditions for stunting.

## Electronic supplementary material

Below is the link to the electronic supplementary material.

Supplementary Material 1

## Data Availability

The data that support the findings of this study are not openly available due to reasons of sensitivity and are available from the corresponding author upon reasonable request.

## List of abbreviations

95% CI: Confidence interval at 95%.
ENSANUT: National Health and Nutrition Survey of Ecuador.
HAZ: Height-for-age Z-scores.
MICS: Multiple Indicator Cluster Survey.
PR: Prevalence ratio.
SD: Standard deviation.
UNICEF: United Nations International Children’s Emergency Fund.
