# Supplementary material for: High prevalence of chronic malnutrition in indigenous children under 5 years of age in Chimborazo-Ecuador: multicausal analysis of its determinants
Source: BMC Public Health. 2022 Oct 28;22:1977. doi: 10.1186/s12889-022-14327-x (PMC9617340; doi:10.1186/s12889-022-14327-x)
Supplement: Supplementary file 1 — Supplementary Material 1 [file 12889_2022_14327_MOESM1_ESM.docx]

**Supplementary material 1.**

**Characteristics of the children included in the study and its association with stunting. Bivariate regression (n=1204)**

|  | **Total** | **Chronic Malnutrition** | ***p* value** | **PR (95% CI) ^†^** | ***p* value** |
| --- | --- | --- | --- | --- | --- |
|  | **N (%) 1099** | **n (%)** |  |  |  |
|  |  |  |  |  |  |
| **SOCIOECONOMIC** |  |  |  |  |  |
| Family income |  |  |  |  |  |
| Q4 | 287 (25.38) | 133 (46.34) | 0.009 | 1.0 |  |
| Q3 | 170 (15.03) | 87 (51.18) |  | 1.1 (0.91-1.33) | 0.312 |
| Q2 | 277 (24.49) | 151 (54.51) |  | 1.17 (0.99-1.38) | 0.053 |
| Q1 | 397 (35.1) | 235 (59.19) |  | 1.27 (1.1-1.48) | 0.001 |
| Mother's schooling level |  |  |  |  |  |
| University/College | 64 (5.8) | 22 (34.38) | <0.001 | 1.0 |  |
| High school | 372 (33.73) | 177 (47.58) |  | 1.38 (0.97-1.97) | 0.073 |
| Primary | 639 (57.93) | 365 (57.12) |  | 1.66 (1.17-2.34) | 0.004 |
| Elementary | 28 (2.54) | 19 (67.86) |  | 1.97 (1.29-3.01) | 0.002 |
| Father's schooling level |  |  |  |  |  |
| University/College | 91 (8.22) | 37 (40.66) | <0.001 | 1.0 |  |
| High school | 422 (38.12) | 204 (48.34) |  | 1.18 (0.91-1.55) | 0.204 |
| Primary | 536 (48.42) | 313 (58.4) |  | 1.43 (1.11-1.85) | 0.006 |
| Elementary/None | 58 (5.24) | 37 (63.79) |  | 1.56 (1.14-2.15) | 0.005 |
| Mother works |  |  |  |  |  |
| No | 632 (54.77) | 319 (50.47) | 0.01 | 1.0 |  |
| Yes | 522 (45.23) | 303 (58.05) |  | 1.15 (1.03-1.27) | 0.01 |
| Parents live together |  |  |  |  |  |
| Yes | 922 (77.94) | 492 (53.36) | 0.397 | 1.0 |  |
| No | 261 (22.06) | 147 (56.32) |  | 1.05 (0.93-1.19) | 0.389 |
| Father lives at home |  |  |  |  |  |
| Yes | 1155 (97.88) | 621 (53.77) | 0.536 | 1.0 |  |
| No | 25 (2.12) | 15 (60) |  | 1.11 (0.80-1.54) | 0.508 |
| Mother lives at home |  |  |  |  |  |
| Yes | 926 (79.15) | 495 (53.46) | 0.327 | 1.0 |  |
| No | 244 (20.85) | 139 (56.97) |  | 1.06 (0.94-1.20) | 0.317 |
| Main roof material |  |  |  |  |  |
| Concrete / slab | 328 (27.84) | 150 (45.73) | 0.002 | 1.0 |  |
| Asbestos (Eternit) | 415 (35.23) | 242 (58.31) |  | 1.27 (1.1-1.47) | 0.001 |
| Zinc | 327 (27.76) | 174 (53.21) |  | 1.16 (0.99-1.35) | 0.057 |
| Tile / straw / other | 108 (9.17) | 66 (61.11) |  | 1.33 (1.1-1.61) | 0.003 |
| Main walls material |  |  |  |  |  |
| Concrete / block | 998 (84.72) | 532 (53.31) | 0.798 | 1.0 |  |
| Asbestos / cement (fibrolit) | 54 (4.58) | 29 (53.7) |  | 1.00 (0.78-1.29) | 0.954 |
| Adobe | 80 (6.79) | 43 (53.75) |  | 1.00 (0.81-1.24) | 0.939 |
| Wood / wattle and daub / mat / other | 46 (3.9) | 28 (60.87) |  | 1.14 (0.89-1.45) | 0.277 |
| Main floor material |  |  |  |  |  |
| Parquet / ceramic / floor tile / marble | 132 (11.21) | 62 (9.81) | 0.003 | 1.0 |  |
| Cement / Wood | 711 (60.36) | 365 (57.75) |  | 1.09 (0.89-1.32) | 0.372 |
| Dirt | 335 (28.44) | 205 (32.44) |  | 1.30 (1.06-1.59) | 0.01 |
| Human development bonus |  |  |  |  |  |
| Yes | 248 (21.07) | 160 (64.52) | <0.001 | 1.0 |  |
| No | 929 (78.93) | 471 (50.7) |  | 0.78 (0.70-0.87) | 0.000 |
| Cook mainly with |  |  |  |  |  |
| Gas / electricity | 980 (83.26) | 507 (51.73) | 0.004 | 1.0 |  |
| Firewood / charcoal | 197 (16.74) | 124 (62.94) |  | 1.21 (1.07-1.37) | 0.002 |
| The water supply is |  |  |  |  |  |
| Permanent | 951 (81.49) | 518 (54.47) | 0.346 | 1.0 |  |
| Irregular | 216 (18.51) | 110 (50.93) |  | 0.93 (0.81-1.07) | 0.358 |
| Do you pay for the water you consume |  |  |  |  |  |
| Yes | 1081 (92.47) | 577 (53.38) | 0.408 | 1.0 |  |
| No | 88 (7.53) | 51 (57.95) |  | 1.08 (0.90-1.30) | 0.387 |
| Water for cooking |  |  |  |  |  |
| Bottled water | 99 (8.4) | 44 (44.44) | 0.122 | 1.0 |  |
| From the public network | 1018 (86.42) | 552 (54.22) |  | 1.22 (0.97-1.53) | 0.087 |
| From the river / other | 61 (5.18) | 36 (59.02) |  | 1.32 (0.97-1.79) | 0.067 |
| Treatment of water for cooking |  |  |  |  |  |
| Boiled | 742 (63.42) | 424 (57.14) | 0.002 | 1.0 |  |
| Chlorine | 92 (7.86) | 51 (55.43) |  | 0.97 (0.79-1.17) | 0.759 |
| Nothing / just filters | 336 (28.72) | 154 (45.83) |  | 0.80 (0.70-0.91) | 0.001 |
| Does the household have electricity |  |  |  |  |  |
| Yes | 1076 (93) | 567 (52.7) | 0.008 | 1.0 |  |
| No | 81 (7) | 55 (67.9) |  | 1.28 (1.09-1.51) | 0.002 |
| Children from 6 to 12 years old that does not go to school |  |  |  |  |  |
| No | 1079 (95.57) | 588 (54.49) | 0.729 | 1.0 |  |
| Yes | 50 (4.43) | 26 (52) |  | 0.95 (0.72-1.25) | 0.736 |
| Receiving remittances |  |  |  |  |  |
| Yes | 37 (3.34) | 16 (43.24) | 0.221 | 1.0 |  |
| No | 1072 (96.66) | 573 (53.45) |  | 1.23 (0.85-1.79) | 0.266 |
|  |  |  |  |  |  |
| **ENVIRONMENTAL** |  |  |  |  |  |
| From where comes the water in the house |  |  |  |  |  |
| Public network | 663 (56.33) | 363 (54.75) | 0.147 | 1.0 |  |
| Public tap | 114 (9.69) | 67 (58.77) |  | 1.07 (0.91-1.27) | 0.41 |
| Another source by pipe | 286 (24.3) | 136 (47.55) |  | 0.86 (0.75-0.99) | 0.049 |
| Well | 14 (1.19) | 9 (64.29) |  | 1.17 (0.78-1.74) | 0.428 |
| River, slope, ditch | 88 (7.48) | 52 (59.09) |  | 1.07 (0.89-1.3) | 0.424 |
| Other | 12 (1.02) | 5 (41.67) |  | 0.76 (0.38-1.49) | 0.427 |
| The water you receive is |  |  |  |  |  |
| By pipeline inside the house | 499 (42.72) | 239 (47.9) | <0.001 | 1.0 |  |
| By pipeline outside the house, but inside the lot | 596 (51.03) | 355 (59.56) |  | 1.24 (1.11-1.39) | <0.001 |
| Piped outside the lot | 41 (3.51) | 13 (31.71) |  | 0.66 (0.41-1.04) | 0.078 |
| No piped water | 32 (2.74) | 21 (65.63) |  | 1.37 (1.04-1.78) | 0.021 |
| The sanitary areas of the dwelling are |  |  |  |  |  |
| Toilet connected to public sewage system | 449 (38.15) | 225 (50.11) | 0.066 | 1.0 |  |
| Toilet connected to septic tank | 366 (31.1) | 192 (52.46) |  | 1.04 (0.91-1.19) | 0.504 |
| Toilet connected to cesspool | 221 (18.78) | 135 (61.09) |  | 1.21 (1.05-1.4) | 0.006 |
| Latrine | 63 (5.35) | 33 (52.38) |  | 1.04 (0.81-1.34) | 0.731 |
| No sanitary area | 78 (6.63) | 47 (60.26) |  | 1.2 (0.98-1.47) | 0.074 |
| How is garbage disposed |  |  |  |  |  |
| Public collection service | 813 (69.37) | 418 (51.41) | 0.081 | 1.0 |  |
| Dumped in the street, stream, river | 10 (0.85) | 6 (60) |  | 1.16 (0.7-1.94) | 0.553 |
| Burned, buried, another | 349 (29.78) | 204 (58.45) |  | 1.13 (1.01-1.27) | 0.023 |
| Overcrowding |  |  |  |  |  |
| No overcrowding | 568 (48.8) | 274 (48.24) | <0.001 | 1 |  |
| Overcrowding | 596 (51.2) | 353 (59.23) |  | 1.22 (1.10-1.36) | <0.001 |
|  |  |  |  |  |  |
| **HEALTHCARE** |  |  |  |  |  |
| Child's health insurance |  |  |  |  |  |
| Yes | 183 (15.42) | 101 (55.19) | 0.635 | 1.0 |  |
| No | 1004 (84.58) | 535 (53.29) |  | 0.96 (0.83-1.11) | 0.63 |
| Number of prenatal checkups |  |  |  |  |  |
| 9 or more | 106 (10.69) | 53 (50) | 0.519 | 1 |  |
| 5 to 8 | 597 (60.18) | 317 (53.1) |  | 1.06 (0.86-1.3) | 0.565 |
| 0 to 4 | 289 (29.13) | 162 (56.06) |  | 1.12 (0.9-1.39) | 0.3 |
| Trimester of pregnancy of the first control |  |  |  |  |  |
| First trimester | 701 (70.81) | 361 (51.5) | 0.088 | 1.0 |  |
| Second or third trimester | 289 (29.19) | 166 (57.44) |  | 1.11 (0.98-1.26) | 0.081 |
| Where did you deliver? |  |  |  |  |  |
| Some health establishment (public or private) | 800 (70.24) | 396 (49.5) | 0.003 | 1.0 |  |
| At home with a midwife, family member or alone | 334 (29.32) | 200 (59.88) |  | 1.21 (1.08-1.35) | 0.001 |
| Other | 5 (0.44) | 4 (80) |  | 1.61 (1.03-2.51) | 0.034 |
| Who cared for you during the delivery |  |  |  |  |  |
| Doctor | 533 (44.79) | 289 (54.22) | <0.001 | 1.0 |  |
| Obstetrician, Nurse, Auxiliary | 278 (23.36) | 118 (42.45) |  | 0.78 (0.66-0.91) | 0.002 |
| Midwife | 182 (15.29) | 111 (60.99) |  | 1.12 (0.97-1.29) | 0.1 |
| Delivery at home | 197 (16.55) | 119 (60.41) |  | 1.11 (0.97-1.27) | 0.123 |
| Delivery type |  |  |  |  |  |
| Caesarean section | 202 (16.86) | 97 (48.02) | 0.082 | 1.0 |  |
| No caesarean section | 996 (83.14) | 545 (54.72) |  | 1.13 (0.97-1.32) | 0.097 |
| Delivery time |  |  |  |  |  |
| Completed | 858 (76.88) | 444 (51.75) | 0.432 | 1.0 |  |
| Postmature | 33 (2.96) | 15 (45.45) |  | 0.87 (0.6-1.28) | 0.503 |
| Premature | 225 (20.16) | 125 (55.56) |  | 1.07 (0.93-1.22) | 0.298 |
| Baby weighted at birth |  |  |  |  |  |
| Yes | 670 (57.76) | 344 (51.34) | 0.003 | 1.0 |  |
| No | 166 (14.31) | 109 (65.66) |  | 1.27 (1.12-1.46) | <0.001 |
| Don't know | 324 (27.93) | 168 (51.85) |  | 1.01 (0.88-1.14) | 0.88 |
| Control of the child after delivery |  |  |  |  |  |
| Yes | 967 (87.2) | 500 (51.71) | 0.069 | 1.0 |  |
| No | 142 (12.8) | 85 (59.86) |  | 1.15 (0.99-1.34) | 0.052 |
| First check-up after birth |  |  |  |  |  |
| Less than 1 week | 290 (26.39) | 140 (48.28) | 0.065 | 1.0 |  |
| 1 week | 201 (18.29) | 106 (52.74) |  | 1.09 (0.91-1.3) | 0.328 |
| 2 or more weeks | 584 (53.14) | 313 (53.6) |  | 1.11 (0.96-1.27) | 0.146 |
| No control | 24 (2.18) | 18 (75) |  | 1.55 (1.19-2.01) | 0.001 |
| Control after mother delivery |  |  |  |  |  |
| Yes | 584 (69.61) | 299 (51.2) | 0.493 | 1.0 |  |
| No | 255 (30.39) | 124 (48.63) |  | 0.94 (0.81-1.10) | 0.498 |
| Mother's first postpartum checkup |  |  |  |  |  |
| Less than 1 week | 307 (29.89) | 166 (54.07) | 0.596 | 1.0 |  |
| 1 week | 113 (11) | 54 (47.79) |  | 0.88 (0.71-1.09) | 0.268 |
| After 1 week | 491 (47.81) | 264 (53.77) |  | 0.99 (0.87-1.13) | 0.933 |
| No control | 116 (11.3) | 58 (50) |  | 0.92 (0.75-1.13) | 0.463 |
| Times the child was hospitalized in the last year |  |  |  |  |  |
| None | 1089 (91.51) | 579 (53.17) | 0.312 | 1.0 |  |
| More than 1 time | 101 (8.49) | 59 (58.42) |  | 1.09 (0.92-1.30) | 0.288 |
| Kid goes to daycare |  |  |  |  |  |
| Yes | 1068 (89.45) | 578 (54.12) | 0.38 | 1.0 |  |
| No | 126 (10.55) | 63 (50) |  | 0.92 (0.76-1.11) | 0.397 |
| Which of the following programs do you attend? |  |  |  |  |  |
| Children's Center of Good Living | 567 (52.6) | 337 (59.44) | 0.001 | 1.0 |  |
| Growing with Our Children | 15 (1.39) | 10 (66.67) |  | 1.12 (0.77-1.61) | 0.537 |
| Initial Education 1 (3-4 years) | 256 (23.75) | 119 (46.48) |  | 0.78 (0.67-0.90) | 0.001 |
| Initial Education 2 (4-5 years) | 240 (22.26) | 115 (47.92) |  | 0.80 (0.69-0.93) | 0.004 |
| Mother’s health insurance |  |  |  |  |  |
| Yes | 171 (14.64) | 90 (52.63) | 0.822 | 1.0 |  |
| No | 997 (85.36) | 534 (53.56) |  | 1.01 (0.87-1.18) | 0.823 |
| Use of birth control |  |  |  |  |  |
| Yes | 641 (55.07) | 342 (53.35) | 0.334 | 1.0 |  |
| No | 481 (51.32) | 252 (52.39) |  | 0.98 (0.87-1.09) | 0.75 |
| Does not know / Does not respond | 42 (3.61) | 27 (64.29) |  | 1.20 (0.95-1.52) | 0.123 |
| Breastfed immediately after birth |  |  |  |  |  |
| Yes | 844 (70.57) | 450 (53.32) | 0.739 | 1.0 |  |
| No | 267 (22.32) | 142 (53.18) |  | 0.99 (0.87-1.13) | 0.97 |
| Does not know / Does not respond | 85 (7.11) | 49 (57.65) |  | 1.08 (0.89-1.31) | 0.428 |
| Child’s health control within 6 months |  |  |  |  |  |
| Twice or more | 593 (51.03) | 337 (56.83) | 0.07 | 1.0 |  |
| Once | 201 (17.3) | 104 (51.74) |  | 0.91 (0.78-1.05) | 0.223 |
| None | 368 (31.67) | 182 (49.46) |  | 0.87 (0.76-0.98) | 0.029 |
| Visited by community care technicians within 6 months |  |  |  |  |  |
| Yes | 232 (40.85) | 137 (59.05) | 0.755 | 1.0 |  |
| No | 336 (59.15) | 194 (57.74) |  | 0.97 (0.84-1.12) | 0.755 |
| Child weighted and measured at the health center within 6 months |  |  |  |  |  |
| Yes | 929 (79.4) | 505 (54.36) | 0.42 | 1.0 |  |
| No | 241 (20.6) | 124 (51.45) |  | 0.94 (0.82-1.08) | 0.429 |
| Receiving iron for preventing anemia |  |  |  |  |  |
| Yes | 404 (35.69) | 232 (57.43) | 0.045 | 1 |  |
| No | 728 (64.31) | 373 (51.24) |  | 0.89 (0.79-0.99) | 0.042 |
|  |  |  |  |  |  |
| **BREASTFEEDING AND CARE PRACTICES** |  |  |  |  |  |
| Up to what age of your child did you breastfed him/her |  |  |  |  |  |
| More than a year | 1046 (88.42) | 556 (53.15) | 0.444 | 1.0 |  |
| From 6 months to a year | 114 (9.64) | 64 (56.14) |  | 1.05 (0.88-1.25) | 0.534 |
| Less than 6 months | 23 (1.94) | 15 (65.22) |  | 1.22 (0.9-1.66) | 0.187 |
| If the child is sick, the father helps you to take care of him |  |  |  |  |  |
| Yes | 926 (81.73) | 493 (53.24) | 0.979 | 1.0 |  |
| No | 207 (18.27) | 110 (53.14) |  | 0.99 (0.86-1.14) | 0.979 |
| To take your kid to a health facility, you ask the father for permission |  |  |  |  |  |
| No | 784 (69.26) | 400 (51.02) | 0.023 | 1.0 |  |
| Yes | 348 (30.74) | 203 (58.33) |  | 1.14 (1.02-1.27) | 0.019 |
| To buy medicines for your kid, you need money from the father |  |  |  |  |  |
| No | 304 (26.97) | 148 (48.68) | 0.068 | 1.0 |  |
| Yes | 823 (73.03) | 451 (54.8) |  | 1.12 (0.98-1.28) | 0.077 |
| What do you use to wash your hands? |  |  |  |  |  |
| Soap | 989 (83.11) | 534 (53.99) | 0.729 | 1.0 |  |
| Something other than soap | 22 (1.85) | 10 (45.45) |  | 0.84 (0.53-1.33) | 0.465 |
| Just water | 179 (15.04) | 96 (53.63) |  | 0.99 (0.85-1.15) | 0.929 |
| Washing fruits before eating |  |  |  |  |  |
| Yes | 1142 (97.03) | 614 (53.77) | 0.544 | 1.0 |  |
| No | 35 (2.97) | 17 (48.57) |  | 0.90 (0.63-1.27) | 0.564 |
| Leave the food to room temperature for 4 hours |  |  |  |  |  |
| Yes | 662 (56.48) | 362 (54.68) | 0.39 | 1.0 |  |
| No | 510 (53.52) | 266 (52.16) |  | 0.95 (0.85-1.06) | 0.392 |
| Is raw food in contact with cooked food |  |  |  |  |  |
| Yes | 736 (63.07) | 395 (53.67) | 0.981 | 1.0 |  |
| No | 431 (36.93) | 231 (53.6) |  | 0.99 (0.89-1.11) | 0.981 |
| Boil the water before drinking it |  |  |  |  |  |
| Yes | 843 (71.93) | 471 (55.87) | 0.012 | 1.0 |  |
| No | 329 (28.07) | 157 (47.72) |  | 0.85 (0.75-0.97) | 0.016 |
| Mother works |  |  |  |  |  |
| Yes | 522 (45.23) | 303 (58.05) | 0.01 | 1.0 |  |
| No | 632 (54.77) | 319 (50.47) |  | 0.86 (0.78-0.96) | 0.01 |
| Father works |  |  |  |  |  |
| Yes | 829 (76.9) | 441 (53.2) | 0.777 | 1.0 |  |
| No | 249 (23.1) | 135 (54.22) |  | 1.01 (0.89-1.16) | 0.776 |
| The father gave you money to support the kid on the last 12 months |  |  |  |  |  |
| Yes | 990 (88.63) | 516 (52.12) | 0.032 | 1.0 |  |
| No | 127 (11.37) | 79 (52.2) |  | 1.19 (1.03-1.38) | 0.019 |
| Children under the age of five on the household |  |  |  |  |  |
| 1 or 2 children | 1130 (96.25) | 604 (53.45) | 0.184 | 1.0 |  |
| 3 or more | 44 (3.75) | 28 (63.64) |  | 1.19 (0.94-1.49) | 0.137 |
| Persons taking care of the child |  |  |  |  |  |
| 3 or more | 276 (23.83) | 140 (50.72) | 0.325 | 1.0 |  |
| 2 persons | 592 (51.12) | 314 (53.04) |  | 1.04 (0.91-1.20) | 0.529 |
| One person | 290 (25.04) | 165 (56.9) |  | 1.12 (0.96-1.30) | 0.143 |
| Daily time to prepare food |  |  |  |  |  |
| More than 120 minutes | 623 (53.29) | 315 (50.56) | 0.02 | 1.0 |  |
| 61 to 119 minutes | 123 (10.52) | 63 (51.22) |  | 1.01 (0.83-1.22) | 0.894 |
| Up to 60 minutes | 423 (36.18) | 250 (59.1) |  | 1.16 (1.04-1.30) | 0.006 |
| Daily time to feed the child |  |  |  |  |  |
| More than 60 minutes | 290 (26.22) | 156 (53.79) | 0.834 | 1.0 |  |
| 31 to 60 minutes | 314 (28.39) | 169 (53.82) |  | 1.00 (0.86-1.16) | 0.994 |
| Up to 30 minutes | 502 (45.39) | 261 (51.99) |  | 0.96 (0.84-1.10) | 0.623 |
| Daily time spent playing with the child |  |  |  |  |  |
| More than 60 minutes | 308 (27.11) | 164 (53.25) | 0.942 | 1.0 |  |
| 31 to 60 minutes | 445 (39.17) | 240 (53.93) |  | 1.01 (0.88-1.15) | 0.853 |
| Up to 30 minutes | 383 (33.71) | 202 (52.74) |  | 0.99 (0.86-1.14) | 0.895 |
| Early initiation of breastfeeding: Children who were breastfed within the first hour of life |  |  |  |  |  |
| Yes - First hour of life | 836 (73.72) | 447 (53.47) | 0.973 | 1.0 |  |
| No - More than one hour of life | 298 (26.28) | 159 (53.36) |  | 0.99 (0.88-1.12) | 0.973 |
| Continued breastfeeding: you continued to breastfeed the child from 6 months of age |  |  |  |  |  |
| Yes | 1160 (97.07) | 620 (53.45) | 0.444 | 1.0 |  |
| No | 35 (2.93) | 21 (60) |  | 1.12 (0.85-1.47) | 0.411 |
| Exclusive breastfeeding: children who were exclusively breastfed before reaching 6 months of age |  |  |  |  |  |
| Yes | 1044 (86.71) | 552 (52.87) | 0.165 | 1.0 |  |
| No | 160 (13.29) | 94 (58.75) |  | 1.11 (0.96-1.28) | 0.146 |
| Introduction to food |  |  |  |  |  |
| At 6 months | 599 (51.86) | 321 (53.59) | 0.728 | 1.0 |  |
| More than 6 months | 429 (37.14) | 226 (52.68) |  | 0.98 (0.87-1.10) | 0.774 |
| Before 6 months | 127 (11) | 72 (56.69) |  | 1.05 (0.89-1.25) | 0.515 |
| Introduction to porridges, fruits, and vegetables (semisolid) |  |  |  |  |  |
| At 6 months | 483 (42.44) | 261 (54.04) | 0.289 | 1.0 |  |
| More than 6 months | 579 (50.88) | 303 (52.33) |  | 0.96 (0.86-1.08) | 0.579 |
| Before 6 months | 76 (6.68) | 47 (61.84) |  | 1.14 (0.94-1.39) | 0.175 |
| Introduction to meat |  |  |  |  |  |
| At 6 months | 943 (83.38) | 501 (53.13) | 0.476 | 1.0 |  |
| More than 6 months | 164 (14.5) | 95 (57.93) |  | 1.09 (0.94-1.25) | 0.238 |
| Before 6 months | 24 (2.12) | 14 (58.33) |  | 1.09 (0.77-1.54) | 0.594 |
| Has parasites - copro |  |  |  |  |  |
| No | 570 (49.31) | 296 (51.93) | 0.135 | 1.0 |  |
| Yes | 586 (50.69) | 330 (56.31) |  | 1.08 (0.97-1.20) | 0.136 |
| Has parasites - copro-concentrate |  |  |  |  |  |
| No | 567 (49.05) | 295 (52.03) | 0.155 | 1.0 |  |
| Yes | 589 (50.95) | 331 (56.2) |  | 1.08 (0.97-1.20) | 0.156 |
| Mother has obesity |  |  |  |  |  |
| Underweight or normal range | 518 (52.32) | 293 (56.56) | 0.062 | 1.0 |  |
| Overweight or obesity | 472 (47.68) | 239 (50.64) |  | 0.89 (0.79-1.00) | 0.063 |
| Dietary diversity in children from 6 to 23 months |  |  |  |  |  |
| Food groups ≥ 4 | 206 (83.4) | 99 (48.06) | 0.626 | 1.0 |  |
| Food groups ≤ 4 | 41 (16.6) | 18 (43.09) |  | 0.91 (0.62-1.32) | 0.636 |
| Is the child being breastfed - children 6 to 23 months |  |  |  |  |  |
| Being breastfed | 142 (56.57) | 67 (47.18) | 0.951 | 1.0 |  |
| Not being breastfed | 109 (43.43) | 51 (46.79) |  | 0.99 (0.76-1.29) | 0.951 |
| Minimum meal frequency in lactating children 6 to 23 months |  |  |  |  |  |
| Above minimum frequency | 114 (87.02) | 56 (49.12) | 0.129 | 1.0 |  |
| It does not reach the minimum frequency | 17 (12.98) | 5 (29.41) |  | 0.59 (0.27-1.28) | 0.187 |
| Minimum meal frequency in non-lactating children 6 to 23 months |  |  |  |  |  |
| Above minimum frequency | 57 (55.88) | 23 (40.35) | 0.278 | 1.0 |  |
| It does not reach the minimum frequency | 45 (44.12) | 23 (45.10) |  | 1.26 (0.82-1.94) | 0.279 |
|  |  |  |  |  |  |
| **BIOLOGICAL CHARACTERISTICS** |  |  |  |  |  |
| Sex |  |  |  |  |  |
| Male | 603 (50.08) | 344 (57.05) | 0.018 | 1.0 |  |
| Feminine | 601 (49.92) | 302 (50.25) |  | 0.88 (0.79-0.97) | 0.018 |
| Age |  |  |  |  |  |
| 0-12 | 61 (5.07) | 21 (34.43) | <0.001 | 1.0 |  |
| 13-24 | 229 (19.02) | 115 (50.22) |  | 1.45 (1-2.11) | 0.045 |
| 25-36 | 334 (27.74) | 215 (64.37) |  | 1.86 (1.31-2.66) | 0.001 |
| 37-48 | 211 (17.52) | 110 (52.13) |  | 1.51 (1.04-2.19) | 0.028 |
| 49-86 | 369 (30.65) | 185 (50.14) |  | 1.45 (1.01-2.08) | 0.041 |
| Mother's age |  |  |  |  |  |
| 13-25 | 477 (41.59) | 234 (49.06) | 0.042 | 1.0 |  |
| 26-35 | 444 (38.71) | 250 (56.31) |  | 1.14 (1.01-1.29) | 0.028 |
| 36 or more | 226 (19.7) | 129 (57.08) |  | 1.16 (1.00-1.34) | 0.041 |
| Mother height |  |  |  |  |  |
| > = 150 cm | 635 (52.74) | 284 (44.74) | <0.001 | 1.0 |  |
| <150 cm | 569 (47.26) | 362 (63.62) |  | 1.42 (1.27-1.58) | <0.001 |
| Size of the child at birth |  |  |  |  |  |
| Very large | 139 (11.58) | 52 (37.41) | <0.001 | 1.0 |  |
| Average size | 756 (63) | 393 (51.98) |  | 1.38 (1.11-1.74) | 0.004 |
| Very small | 266 (22.17) | 175 (65.79) |  | 1.75 (1.39-2.21) | <0.001 |
| Don't know / don't remember | 39 (3.25) | 24 (61.54) |  | 1.64 (1.18-2.28) | 0.003 |
| Number of children by mother |  |  |  |  |  |
| 1-2 | 683 (58.58) | 326 (47.73) | <0.001 | 1.0 |  |
| 3-4 | 315 (27.02) | 180 (57.14) |  | 1.19 (1.05-1.35) | 0.004 |
| ≥5 | 168 (14.41) | 116 (69.05) |  | 1.44 (1.27-1.64) | <0.001 |
| Diarrhea in the past 6 months |  |  |  |  |  |
| None | 529 (43.97) | 269 (50.85) | 0.001 | 1.0 |  |
| 1 to 2 times | 371 (30.84) | 183 (49.33) |  | 0.97 (0.84-1.11) | 0.654 |
| More than 2 times | 277 (23.03) | 178 (64.26) |  | 1.26 (1.11-1.42) | <0.001 |
| Don't know | 26 (2.16) | 16 (61.54) |  | 1.21 (0.88-1.65) | 0.236 |
| Delivery time |  |  |  |  |  |
| Completed | 858 (76.88) | 444 (51.75) | 0.432 | 1.0 |  |
| Postmature | 33 (2.96) | 15 (45.45) |  | 0.87 (0.60-1.28) | 0.503 |
| Premature | 225 (20.16) | 125 (55.56) |  | 1.07 (0.93-1.22) | 0.298 |
| Kid weighted at birth |  |  |  |  |  |
| Yes | 670 (57.76) | 344 (51.34) | 0.003 | 1.0 |  |
| No | 166 (14.31) | 109 (65.66) |  | 1.27 (1.12-1.46) | <0.001 |
| Don't know | 324 (27.93) | 168 (51.85) |  | 1.00 (0.88-1.14) | 0.88 |
| Birth interval |  |  |  |  |  |
| Over 4 years | 341 (50.37) | 184 (53.96) | 0.205 | 1.0 |  |
| 2 to 4 years | 238 (35.16) | 145 (60.92) |  | 1.12 (0.98-1.30) | 0.092 |
| Less than 2 years | 98 (14.48) | 59 (60.2) |  | 1.11 (0.92-1.34) | 0.255 |
| Anemia |  |  |  |  |  |
| No | 665 (77.61) | 341 (52.06) | 0.08 | 1.0 |  |
| Yes | 189 (22.39) | 112 (59.26) |  | 1.13 (0.99-1.30) | 0.068 |
| Times you took the child to the health center in the last 6 months |  |  |  |  |  |
| More than twice | 271 (23.02) | 167 (61.62) | 0.003 | 1.0 |  |
| 1 to 2 times | 480 (40.78) | 260 (54.17) |  | 0.87 (0.77-0.99) | 0.043 |
| None | 426 (36.19) | 206 (48.36) |  | 0.78 (0.68-0.89) | <0.001 |
| How many times has the child been hospitalized in the last year |  |  |  |  |  |
| None | 1089 (91.51) | 579 (53.17) | 0.065 | 1.0 |  |
| 1 to 2 times | 91 (7.65) | 50 (54.95) |  | 1.03 (0.85-1.25) | 0.74 |
| More than twice | 10 (0.84) | 9 (90) |  | 1.69 (1.36-2.09) | <0.001 |
| Times the child has had parasites in the past 6 months |  |  |  |  |  |
| None | 576 (70.67) | 277 (48.09) | <0.001 | 1.0 |  |
| 1 to 2 times | 223 (27.36) | 139 (62.33) |  | 1.29 (1.13-1.48) | <0.001 |
| More than twice | 16 (1.96) | 14 (87.5) |  | 1.81 (1.48-2.23) | <0.001 |
| Times child has had parasites in the last year |  |  |  |  |  |
| None | 621 (68.62) | 300 (48.31) | <0.001 | 1.0 |  |
| 1 to 2 times | 262 (28.95) | 159 (60.69) |  | 1.25 (1.10-1.42) | <0.001 |
| More than twice | 22 (2.43) | 17 (77.27) |  | 1.59 (1.25-2.03) | <0.001 |
| Weight at birth |  |  |  |  |  |
| More than 4 kg | 10 (2.89) | 4 (40) | 0.083 | 1.0 |  |
| Between 2.5 and 4 kg | 303 (87.57) | 142 (46.86) |  | 1.17 (0.54-2.52) | 0.687 |
| Less than 2.5 kg | 33 (9.54) | 22 (66.67) |  | 1.66 (0.75-3.709) | 0.209 |
| Size at birth |  |  |  |  |  |
| 46 cm or more | 263 (79.7) | 117 (44.49) | 0.008 | 1.0 |  |
| Less than 46 cm | 67 (20.3) | 42 (62.69) |  | 1.40 (1.12-1.77) | 0.003 |

^†^PR (95% CI)= Prevalence Ratio and 95% Confidence Interval
